# Supplementary material for: Discrepancies between FDA documents and ClinicalTrials.gov for Orphan Drug-related clinical trial data
Source: PLOS Glob Public Health. 2022 Apr 22;2(4):e0000261. doi: 10.1371/journal.pgph.0000261 (PMC10021800; doi:10.1371/journal.pgph.0000261)
Supplement: Attachment — Submitted filename: Response to reviewers.doc [file pgph.0000261.s006.doc]

**2. We have noticed that you have uploaded supporting information but you have not included a list of legends. Please add a full list of legends for all supporting information files (including figures, table and data files) after the references list.**

**Response**: The legends were included in the main document. However the legends of the sub-sections were only in each supplementary file. We have now included them in the main manuscript file, after the references list.

- - - - - - - - - - - - - - - - - - - - - - - - - - - - - - - - - - - -

**Reviewer #1**:

**Major issues**

R1.1 In Materials and Methods, the authors presented a very detailed description for the selection of the registries to evaluate, including numbers. However, this may be better located as part of the results.

**Response**: In terms of registries, our manuscript is only concerned with ClinicalTrials.gov, the major public registry of the US. We spent considerable time trying to see which portions of the Materials and Methods could be moved to Results. However, there were many steps of filtering, before we zeroed in on the trials that were to be analyzed. Those flitering steps appear to us to be more of Methods than Results. Also, we are apprehensive that if we move several paragraphs from Methods to Results, one of the other reviewers may ask us to move them back to Methods again. So, we hope it is alright if we leave the Materials and Methods section as it is.

- - - - - - - - - - - - - - - - - - - - - - - - - - - - - - - - - - - -

R1.2 Although the authors did a good job explaining the study methodology, adding some information would be helpful to better understand the work.

**Response**: We have done this in our responses to the other queries, listed by all the reviewers, below.

- - - - - - - - - - - - - - - - - - - - - - - - - - - - - - - - - - - -

R1.3 For example, how exactly they did the comparison between different files, if it was by software or manually.

**Response**: There was no direct comparison between files. Data was extracted from the MedRs and from CTG, and then manual comparisons were made of the data in ‘2_Master Sheet’ of S2 File.

- - - - - - - - - - - - - - - - - - - - - - - - - - - - - - - - - - - -

R1.4 Why they chose studies approved after 2008.

**Response**: The following explanation has been included in the manuscript now, at the start of the Discussion:

We chose to work with drugs approved after 2008 due to the regulations in force at the time. The United States Food and Drug Administration (FDA) Amendments Act of 2007 (U.S. Public Law 110-85), was signed into law on 27 September 2007 (<https://www.fda.gov/regulatory-information/selected-amendments-fdc-act/food-and-drug-administration-amendments-act-fdaaa-2007>). This Law mandated the registration of ‘applicable clinical trials’ (<http://frwebgate.access.gpo.gov/cgi-bin/getdoc.cgi?dbname=110_cong_public_laws&docid=f:publ085.110.pdf>), that is trials - other than Phase 1 trials - used to support the application to the FDA for a new drug’s approval, in a publically accessible trial registry. Although the law came into force in September 2007, we left ample margin, and examined drugs approved after 2008, to maximize the number of drugs whose trials would be available in ClinicalTrials.gov

- - - - - - - - - - - - - - - - - - - - - - - - - - - - - - - - - - - -

R1.5 The reasons for limiting the number of diseases or trials to review the registries and some definitions like “pivotal studies”, these two aspects are presented in the discussion, however, it would be useful to the readers to know them since the methods.

**Response**: Pivotal studies have now been described in Materials and Methods. Also, there has been an elaboration of why we have focussed on RDs/ODs in the Introduction..

- - - - - - - - - - - - - - - - - - - - - - - - - - - - - - - - - - - -

R1.6 A more detailed definition of N1 and N2.

**Response**: This has been done.

- - - - - - - - - - - - - - - - - - - - - - - - - - - - - - - - - - - -

**Minor issues**

R1.7 While the abstract provides the reader with an overview of the work, some information about the methodology is missing, specifically how the comparison was made between the different registries.

**Response**: As mentioned above, we have used data only from the ClinicalTrials.gov registry in this study. However, we have added a line in the Abstract about matching Study identifiers in MedR with trial IDs, Other ID and Acronyms in ClinicalTrials.gov.

- - - - - - - - - - - - - - - - - - - - - - - - - - - - - - - - - - - -

R1.8 As there was a long cascade to choose the final registries to review, it would be useful to the reader to **have a flow chart** with the numbers that lead to the final number, showing the selection criteria at each stage.

**Response**: This has been done.

- - - - - - - - - - - - - - - - - - - - - - - - - - - - - - - - - - - -

R1.9 Although the intention of the tables is to show the findings in a comprehensive manner, tables 2 and 4 do not add much to the description made by the authors in results section. These descriptions can be summarized by presenting the most relevant results.

**Response**: The reviewer appears to be asking us to delete Tables 2 and 4, and just highlight the main points in the text. However, since R2.2 asks for some changes to Tables 2 and 5, we are in a quandry about whether or not to delete Tables 2 and 4. Also, in comment R1.10, there is a request for changes around Table 2. We have therefore retained Tables 2 and 4, but acted upon the suggestions in R1.10 and R2.2.

- - - - - - - - - - - - - - - - - - - - - - - - - - - - - - - - - - - -

R1.10 The description of table 2 began before the table was placed, however after the table there is a complementary description, with all the information included in the table. This description could be summarized and presented before the table to give a better flow of the information presented.

**Response**: This has been done.

- - - - - - - - - - - - - - - - - - - - - - - - - - - - - - - - - - - -

R1.11 It would be useful to the readers if the authors include the description of N1 and N2 in the table captions, as well as the description of abbreviations used in the tables.

**Response**: This has been done.

- - - - - - - - - - - - - - - - - - - - - - - - - - - - - - - - - - - -

**Reviewer #2**: I have put my comments in the attached file. I hope the authors find them useful.

**Response**: We have transferred all of those comments to this document, and numbered them.

- - - - - - - - - - - - - - - - - - - - - - - - - - - - - - - - - - - -

R2.1

Most of my comments have to do with clarity and justification for various decisions the authors have made (e.g., the date range, the outcomes). I think the fact the authors could not find CTG data for 200 of the 422 trials is an important one, even if the attributes were to match closely for the 202 trials.

**Response**: We have included the following lines in the Discussion now: There are various possible reasons why we could not find a trial in CTG. (i) the studies may have run before CTG was established. (ii) Phase 1 trials were not required to be registered until the Final Rule of 2017. (iii) In the UK, an audit has found that 12% of trials haven’t been registered (Research Transparency Annual Report_2021) and a certain fraction of trials may remain unregistered in the US as well. (iv) Finally, it is possible that we were unable to identify the CTG match of some studies although such a match did exist.

- - - - - - - - - - - - - - - - - - - - - - - - - - - - - - - - - - - -

R2.2. In Tables 2 and 5, it would be better to break down rows 2 and 3 (the ones about N1 and N2) into N1>N2 and N1

**Response**: Although this comment was incomplete, we have guessed that the reviewer wished us to give the break up of ‘N2 within +/– 5% of N1’ and ‘N2 not within +/– 5% of N1’ for Tables 2 and 5. This has been done.

- - - - - - - - - - - - - - - - - - - - - - - - - - - - - - - - - - - -

[**The rest of the comments in Reviewer 2’s section are taken from the manuscript, which he/she had annotated with queries.**]

**R2.3 Title: For orphan drug-related clinical trial data, discrepancies between FDA documents and ClinicalTrials.gov**

**Comment:** Consider changing the title to “Discripancies between FDA Documents and ClinicalTrials.gov for Orphan Drug-Related Clinical Trial Data”

**Response**: This has been done.

**- - - - - - - - - - - - - - - - - - - - - - - - - - - - - - - - - - - -**

**R2.4 Row 32**

**Comment:** Provide an example or two

**Response**: We have provided two RDs, and two ODS for these RDs, in the Introduction. We felt that it fit better in the Introduction than in the Abstract.

**- - - - - - - - - - - - - - - - - - - - - - - - - - - - - - - - - - - -**

**R2.5 Row 57**

**Comment:** Minor: Need to explain how. It would also be good to cite other studies to support these claims, if possible.

**Response**: These are well known reasons for the importance of trial registries. Suitable references have been added.

**- - - - - - - - - - - - - - - - - - - - - - - - - - - - - - - - - - - -**

**R2.6 Row 60**

Comment: Minor: It appears to me that some of these are more important reasons for why trials should be registered than the reasons above. Consider mentioning these first.

**Response**: Two of the three reasons were the basis for calls for setting up registries in the first place, in the 1970s (Dickersin_Rennie reference). Once the registries were set up, researchers used the data in them to conduct other analyses. Therefore, we have not changed the sequence of reasons for why trials should be registered.

**- - - - - - - - - - - - - - - - - - - - - - - - - - - - - - - - - - - -**

**R2.7 Row 66**

Comment: Minor: Consider using data as plural, throughout the manuscript.

**Response**: This has been done.

**- - - - - - - - - - - - - - - - - - - - - - - - - - - - - - - - - - - -**

**R2.8 Row 71**

Comment: Either “accessible to” or “accessed by”

**Response**: This has been done. We have stuck to ‘accessible to’.

**- - - - - - - - - - - - - - - - - - - - - - - - - - - - - - - - - - - -**

**R2.9 Row 73**

Comment: researchers

**Response**: Although ‘trialists’ is an acceptable term in the literature, we have made the requested change.

**- - - - - - - - - - - - - - - - - - - - - - - - - - - - - - - - - - - -**

**R2.10 Row 75**

Comment: Not clear what this means

**Response**: We have deleted the phrase, since the sentence is clearer without it.

**- - - - - - - - - - - - - - - - - - - - - - - - - - - - - - - - - - - -**

**R2.11 Row 77**

Comment: Awkward wording (by whom?)

**Response:** The sentence has been modified suitably.

**- - - - - - - - - - - - - - - - - - - - - - - - - - - - - - - - - - - -**

**R2.12 Row 79**

Comment: Awkward wording. Consider removing this phrase.

**Response**: We wished to introduce the concepts of rare diseases and orphan drugs. We have modified the first sentence, and hope it does the job better.

**- - - - - - - - - - - - - - - - - - - - - - - - - - - - - - - - - - - -**

**R2.13 Row 81**

Comment: Diseases?

**Response**: We didn’t want to over-use ‘diseases’ and therefore used ‘maladies’. We hope that it is acceptable if we leave it as it is.

**- - - - - - - - - - - - - - - - - - - - - - - - - - - - - - - - - - - -**

**R2.14 Row 82**

Comment: Because the conditions are rare? Explain.

**Response:** Yes. We have clarified this.

**- - - - - - - - - - - - - - - - - - - - - - - - - - - - - - - - - - - -**

**R2.15 Row 83**

Comment: Aren’t these common to non-rare diseases as well? Clarify.

**Response:** We have reworded this sentence.

**- - - - - - - - - - - - - - - - - - - - - - - - - - - - - - - - - - - -**

**R2.16 Row 84**

Comment: Unclear what the authors mean here

**Response:** An explanation and a new reference have been added.

**- - - - - - - - - - - - - - - - - - - - - - - - - - - - - - - - - - - -**

**R2.17 Row 90**

Comment: Doesn’t the FDA ask for trial registry data when it evaluates a drug? Please confirm.

**Response:** The FDA asks for trial data, but this data comes directly from the sponsor, not from the registry. Independent of its submission to the FDA, the sponsor submits data to the registry. As such, there are two separate pathways of (and destinations for) data submission, and that may be one of the reasons that discrepancies in the data arise.

**- - - - - - - - - - - - - - - - - - - - - - - - - - - - - - - - - - - -**

**R2.18 Row 93**

Comment: Consider changing this word

**Response:** There are several papers/documents in the literature that call for audits of trial data, by, for instance, Prof. Ben Goldacre, at the University of Oxford (Goldacre B (2015) How to Get All Trials Reported: Audit, Better Data, and Individual Accountability. PLoS Med 12(4): e1001821. doi:10.1371/journal.pmed.1001821) and the UK House of Commons (House of Commons Science and Technology Committee. Research integrity: clinical trials transparency. HC1480, 2018.). Therefore we have used the word ‘audit’ in various places in the manuscript. Nevertheless, in response to this request, we have changed it here to limited audit.

**- - - - - - - - - - - - - - - - - - - - - - - - - - - - - - - - - - - -**

**R2.19 Row 95**

Comment: MAJOR: what was the rationale for using these factors?

**Response:** We compared details of (i) phase, (ii) enrollment, and (iii) enrollment attribute (‘anticipated’ or ‘actual’) from the two sources. There is such a strong need for various types of audits of clinical trial data that there has been an assessment of which types of audits require specialist skills and which do not (House of Commons Science and Technology Committee. Research integrity: clinical trials transparency. HC1480, 2018.). We are a team of non-medicos, and we chose to audit fields for which there is no need for specialist medical judgment. The phase and enrollment are fundamental aspects of any trial, and the enrollment attribute is a necessary qualifier of the enrollment number. At a broader level, our manuscript makes the point that if even these basic numbers do not match perfectly, then other fields may not as well. Our paper merely reiterates what others have found, that more care needs to be taken while submitting information to the FDA, and to CTG, and to keeping the data in the latter database up-to-date.

**- - - - - - - - - - - - - - - - - - - - - - - - - - - - - - - - - - - -**

**R2.20 Row 97**

Comment: Please explain what these are, and why you focus on these in your subgroup analysis

**Response:** In the Materials and Methods section we have now explained what pivotal trials are and why we focus on them.

**- - - - - - - - - - - - - - - - - - - - - - - - - - - - - - - - - - - -**

**R2.21 Row 101**

Comment: Please be consistent with tense throughout the manuscript

**Response:** This has been done.

**- - - - - - - - - - - - - - - - - - - - - - - - - - - - - - - - - - - -**

**R2.22 Row 110**

Comment: Awkward usage. Consider editing this sentence, or breaking into two

**Response:** We have broken the sentence into two.

**- - - - - - - - - - - - - - - - - - - - - - - - - - - - - - - - - - - -**

**R2.23 Row 126**

Comment: MAJOR: What was the rationale for limiting the study to this period?

**Response:** Please see our response to query R1.4 (by reviewer 1). This provides the rationale for why we started with drugs approved after 2008. We initiated the study on 18 April 2020, and therefore 17 April 2020 was the last date for an OD to be approved, to be included in our study.

**- - - - - - - - - - - - - - - - - - - - - - - - - - - - - - - - - - - -**

**R2.24 Row 152**

Comment: Why? Please explain.

**Response:** Occasionally a ‘pivotal’ trial was mentioned not in the Table, but elsewhere in the MedR. Since pivotal trials are centrally important to the New Drug Application being submitted to the FDA, we felt justified in including it in the analysis even though it was not listed in the Table.

**- - - - - - - - - - - - - - - - - - - - - - - - - - - - - - - - - - - -**

**R2.25 Row 155**

Comment: Please explain the rationale for these decisions.

**Response:** These trials did not have trial IDs. Therefore it would have been extremely challenging, if not impossible, to find them in CTG.

**- - - - - - - - - - - - - - - - - - - - - - - - - - - - - - - - - - - -**

**R2.26 Row 159**

Comment: When I read the manuscript, it did not feel like putting N1 and N2 instead of the description simplified anything. Consider just calling enrolment in CTG, enrolment in FDA, etc.

**Response:** N1 and N2 occur many times in the manuscript. As such, we felt that an abbreviation may make it easier for the reader. Further, an abbreviation is easier to use in the tables. So, if the Reviewer agrees, we would like to continue to use N1 and N2.

**- - - - - - - - - - - - - - - - - - - - - - - - - - - - - - - - - - - -**

**R2.27 Row 174**

Comment: Not sure if you need to put these details, as most readers can infer it without reading this sentence

**Response:** We spent some time debating this point. Although we could have deleted one sentence, since more clarity may be better than less clarity, we thought of leaving it in, in case the Reviewer is ok with that.

**- - - - - - - - - - - - - - - - - - - - - - - - - - - - - - - - - - - -**

**R2.28 Row 186**

Comment: MAJOR: Again, it is not at all clear why this outcome is important. Please explain.

**Response:** We have added an explanation early in the Discussion.

**- - - - - - - - - - - - - - - - - - - - - - - - - - - - - - - - - - - -**

**R2.29 Row 192**

Comment: What does “these” refer to here?

**Response:** This has been clarified.

**- - - - - - - - - - - - - - - - - - - - - - - - - - - - - - - - - - - -**

**R2.30 Table 1**

Comment: Not sure why the authors did not search this information by going through the document, since these are only two studies.

**Response:** The two documents concern Adcirca (97 pages long, <https://www.accessdata.fda.gov/drugsatfda_docs/nda/2009/022332s000_MedR.pdf>) and Tyvaso (141 pages long, <https://www.accessdata.fda.gov/drugsatfda_docs/nda/2009/022387s000MedR.pdf>). Both are older documents, dated 2009, that is the earliest year of our study. The documents cannot even be searched with a ‘find’ function. As such, we did not have the confidence to be sure that we could manually search 238 pages accurately.
**- - - - - - - - - - - - - - - - - - - - - - - - - - - - - - - - - - - -**

**R2.31 Table 2**

**R2.31a** Comment: MAJOR: Why is N1 > N2 a problem? It is possible that researchers decided to increase the sample size after they registered the study in CTG.

**Response:** If so, they should have gone back and modified N2 in CTG. Registry data is a form of public accountability, and it is important that data in trial registries are accurate, comprehensive and up-to-date.

**- - - - - - - - - - - - - - - - - - - - - - - - - - - - - - - - - - - -**

**R2.31b** Alternatively, why is N2>N1 a problem? Could it be that efficacy was established before the sample size envisioned in the CTG was reached? In that case, N2 being greater than N1 does not seem like a problem.

**Response:** Same response as in R2.31a

**- - - - - - - - - - - - - - - - - - - - - - - - - - - - - - - - - - - -**

**R2.31c** Comment: Please see comment immediately above this.

**Response:** Same response as in R2.31a

**- - - - - - - - - - - - - - - - - - - - - - - - - - - - - - - - - - - -**

**R2.31d** Comment: MAJOR: if the trial is ongoing, the mismatch between MedR and CTG is inflated. Consider removing these trials from the comparison.

**Response:** We found just three trials listed as ‘ongoing’ in the MedRs. We describe these cases here:

1. V00400 SB 3 01 (Study 301), in MedR: No match in CTG, and therefore not part of the study.

2. AT1001-42 in MedR, NCT02194985, in CTG. This trial was one of 12 that we excluded on sheet S4a(i).

3. GBT440-007 in MedR, NCT02850406, in CTG. This trial was one of 12 that we excluded on sheet S4a(i).

In order to prevent confusion, we have removed the last column of Table 2, and the asterisk comment.

**- - - - - - - - - - - - - - - - - - - - - - - - - - - - - - - - - - - -**

**R2.31e** Comment: In that case, consider doing all the comparisons on the 200 trials.

**Response:** Based on our response to the comment above, this comment may not be relevant.

**- - - - - - - - - - - - - - - - - - - - - - - - - - - - - - - - - - - -**

**R2.32 Row 245**

Comment: In this section, please make sure to:

**R2.32a** Summarize your key findings

**Response:** This has been done.

**- - - - - - - - - - - - - - - - - - - - - - - - - - - - - - - - - - - -**

**R2.32b** Possible explanation for your findings – are researchers/drug companies purposefully not registering their trials (if so, what may be the reason)? What can help explain the differences in enrolment numbers?

**Response:** This has been explained in the Discussion.

**- - - - - - - - - - - - - - - - - - - - - - - - - - - - - - - - - - - -**

**Reviewer #3**:

Below are few suggestions to further strengthen the manuscript:

**- - - - - - - - - - - - - - - - - - - - - - - - - - - - - - - - - - - -**

R3.1. Though the authors have defined the term “rare disease”, but would be better if they can also share few examples.

**Response:** This has been done in the Introduction.

**- - - - - - - - - - - - - - - - - - - - - - - - - - - - - - - - - - - -**

R3.2. Under the discussion section, the authors have highlighted “Although the quality of data in CTG has received considerable attention, FDA documents have not been analyzed as much. This study reinforces the idea that, periodically, trial data from multiple 306 sources must be compared to ensure consistency.” The authors could also comment on how this exercise and audit could be streamlined through the use of technology and artificial intelligence. For instance, development of databases which can collate information from different sources, prepares the audit report and highlight the gaps.

**Response:** This has been done in the Discussion.

**- - - - - - - - - - - - - - - - - - - - - - - - - - - - - - - - - - - -**

**Reviewer #4**: General comments:

R4.1. The paragraphs don't flow together very well, so I suggest trying to link them together a bit better. The introduction section is a bit disconnected. I would also suggest having a colleague (who isn't working on this project) proofread to help improve how the text reads, since it is a bit hard to follow in places.

**Response:** This has been done.

**- - - - - - - - - - - - - - - - - - - - - - - - - - - - - - - - - - - -**

R4.2 I think some terms need defining more explicitly in the text for those who aren't completely familiar with the language of trials.

**Response:** This has been done.

**- - - - - - - - - - - - - - - - - - - - - - - - - - - - - - - - - - - -**

R4.3 There are too many acronyms for the text to be easily readable, so I suggest spelling some of them out that are actual expressions, like study identifier , orphan drug, or rare disease. I would also move the abbreviations section above the references.

**Response:** We have retained certain standard acronyms such as FDA, CTG, RD and OD. However we have replaced three acronyms (for Orange Book, for the International Committee of Medical Journal Editors, and for study identifier) that are either non-standard or that occur infrequently in the text.

**- - - - - - - - - - - - - - - - - - - - - - - - - - - - - - - - - - - -**

R4.4 I would also suggest introducing your research aim/question more explicitly, and also putting the 'why' in here (same paragraph). Then refer back to this in the conclusions, since it's not very clear what question you answered.

**Response:** This has been done.

**- - - - - - - - - - - - - - - - - - - - - - - - - - - - - - - - - - - -**

R4.5 Further, I think the materials and methods section needs to be revised to make clearer what you did. S1 is quite clear, so I suggest making this into a flow diagram and making it figure 1. For the text I suggest the following order:

**Response:** A flowchart has been created.

**- - - - - - - - - - - - - - - - - - - - - - - - - - - - - - - - - - - -**

1. Which datasets did you obtain? What did they contain, in terms of variables and why did you need them? (i.e. FDAOD, New Drug Application, Drugs@FDA (?), Orange Book?, MedRs, CTG (?))

**Response:** This has been done. Table 1 has been added in Materials and Methods.

**- - - - - - - - - - - - - - - - - - - - - - - - - - - - - - - - - - - -**

2. How did you extract the data? If it was with a script, what was the script scripted in, and how did it work? Inclusion/exclusion criteria here.

**Response:** CTG data was extracted via CTG’s API using rclinicaltrials package (currently archived in CRAN, at https://cran.r-project.org/) in R scripting. Any other use of scripts was only for preliminary work. All final data was both extracted and verified manually.

**- - - - - - - - - - - - - - - - - - - - - - - - - - - - - - - - - - - -**

3. How did you combine the data together? If into one dataset, how did this dataset look? A table of variables/structure of the final dataset could help here.

**Response:** We did not fully understand the query. The reviewer may please take a look at the sheet ‘2_Mastersheet’ in S2 File.xlsx.

**- - - - - - - - - - - - - - - - - - - - - - - - - - - - - - - - - - - -**

R4.6 To improve the paper, I would suggest doing some simple statistical tests to compare the pivotal trials and all trial datasets. However, the pivotal trials are contained in the all trials dataset (as far as I understand), so you cannot easily draw comparisons between the two groups since they're not mutually exclusive. You can't draw strong conclusions about the accuracy of records between the two groups without this kind of simple analysis. I think the paper would be a lot stronger.

**Response:** We discussed this point with a mathematician. His point was that since we are simply comparing the two registries with zero permissible room for error, there is no source of randomness in the data. This precludes implementation of any statistical test. In other words, the two sets of data must match with no room for error. The presence of any deviation points to evidence of misreporting. Accordingly, we have not carried out any statistical tests.

**- - - - - - - - - - - - - - - - - - - - - - - - - - - - - - - - - - - -**

R4.7 New information shouldn't be introduced in the discussion. Move most of it to the introduction but refer back to it in the discussion so as to engage with the literature.

**Response:** We have done this. Line 255: ‘Since more of the ODs…’ has been merged with line 131.

**- - - - - - - - - - - - - - - - - - - - - - - - - - - - - - - - - - - -**

Specific comments:

R4.8 pg 4, line 75 : What is the Final Rule of 2017?

**Response:** This has been spelled out.

**- - - - - - - - - - - - - - - - - - - - - - - - - - - - - - - - - - - -**

R4.9 pg 4, lines 92-96. "For a given drug, we examined whether each relevant trial listed in the FDA document was also registered with CTG. If so, we compared details of (i) phase, (ii) enrollment, and (iii) enrollment attribute (‘anticipated’ or ‘actual’) from the two sources. We did this for all trials, and separately for the sub-set of pivotal trials."

This should go in the methods and not the introduction.

**Response:** This has been done.

**- - - - - - - - - - - - - - - - - - - - - - - - - - - - - - - - - - - -**

R4.10 I would also suggest introducing your research aim/question more explicitly, and also putting the 'why' in here (same paragraph)

**Response:** This has been done.

**- - - - - - - - - - - - - - - - - - - - - - - - - - - - - - - - - - - -**

R4.11 pg 4 line 101: Please cite where the data is actually obtained from: not just 'publicly available information' but the actual links and names of the datasets. Also this should go in your data availability statement. This section duplicates some effort in the following subsection, so it may be sensible to cut it out entirely or revise to cite the datasets.

**Response**: We started out with an introductory ‘publicly available information’ statement. The URLs are available from the links that are listed as references 28–31. We have added a line to this effect. As such, we feel that it is not a duplication, just an introduction followed by specific details. We hope that the addition of the references in the introductory comments is acceptable.

**- - - - - - - - - - - - - - - - - - - - - - - - - - - - - - - - - - - -**

R4.12 pg 4 line 102: Please go into slightly more detail about the structure of your discussions to resolve discrepancies.

**Response:** We have encountered the phrase ‘settled by discussion’ quite often in our reading of the literature. As far as we can recall, no further details were provided in any case. In order to illustrate this, we went through many of our pdfs, to find such cases. We enclose details of the three cases that we found:

Prof. Ben Goldacre, of Oxford University: 2019 paper. COMPare: a prospective cohort study correcting and monitoring 58 misreported trials. doi: 10.1186/s13063-019-3173-2.

Prof. Jennifer Miller, initially at New York University: 2015 paper. Clinical trial registration, reporting, publication… h[ttp://dx.doi.org/10.1136/bmjopen-2015-009758](http://dx.doi.org/10.1136/bmjopen-2015-009758)

Prof. Jennifer Miller, then at Yale University: 2019 paper. Sharing of clinical trial data and results reporting… doi: 10.1136/bmj.l4217.

We could probably convene a meeting of all the authors (each now working in a different institution) and try to recall the various discrepancies that were resolved, but we hope it is acceptable if we don’t do that.

**- - - - - - - - - - - - - - - - - - - - - - - - - - - - - - - - - - - -**

R4.13 pg 5 lines 105-107: How did you define what orphan drugs are? Was it your own definition of something from the FDA?

**Response:** We considered only those drugs as ODs which had recieved orphan drug designation and a subsequent approval for an orphan indication from the FDA and are listed in the FDA Orphan Drug database. We felt that the wording in lines 105–107 should suffice, but if required we will clarify this further in the manuscript.

**- - - - - - - - - - - - - - - - - - - - - - - - - - - - - - - - - - - -**

R4.14 pg 5 lines 107-108: What details are in the database? A list of variables would be helpful here. (maybe in table form?)

**Response:** The list of variables is now provided in S1a File.

**- - - - - - - - - - - - - - - - - - - - - - - - - - - - - - - - - - - -**

R4.15 pg 5 lines 113-115: How do you determine 'orphan indication'? How does this relate to 'orphan drugs'?

**Response:** An ‘indication’ refers to the precise condition for which the drug is approved. Even a drug approved as an orphan drug could be for various rare/orphan ‘indications’. We identified each ‘orphan indication’ as one for which a drug had received an orphan designation. These indications are listed in the FDAOD database for each OD.

**- - - - - - - - - - - - - - - - - - - - - - - - - - - - - - - - - - - -**

R4.16 pg 5 lines 120-122: The 'single approval list' comes from the FDAOD database, I'm assuming? It's not clear here, so I would make it explicit which list is from which dataset.

**Response:** This has been done.

**- - - - - - - - - - - - - - - - - - - - - - - - - - - - - - - - - - - -**

R4.17 pg 5 line 125: Why did you choose to study those approved after 2008? What's the rationale?

**Response:** This is now covered in the Discussion. Please see details in our response to R1.4 as well.

**- - - - - - - - - - - - - - - - - - - - - - - - - - - - - - - - - - - -**

R4.18 pg 6 line 134-136: The info provided in FDAOD should go with the information about the dataset itself.

**Response:** We have moved that line suitably.

**- - - - - - - - - - - - - - - - - - - - - - - - - - - - - - - - - - - -**

R4.19 pg 6 line 140: Indication here refers to whether or not the trial was for an orphan drug?

**Response:** An ‘indication’ refers to the precise condition for which the drug is approved. Even a drug approved as an orphan drug could be for various rare/orphan ‘indications’.

**- - - - - - - - - - - - - - - - - - - - - - - - - - - - - - - - - - - -**

R4.20 pg 6 lines 145-146: I'm not sure what you're trying to say here: what script did you use for data extraction, what exactly was extracted, and why then did you just extract it manually anyways (so you didn't use a script?)?

**Response**: CTG data was extracted via CTG’s API using rclinicaltrials package (currently archived in CRAN, at https://cran.r-project.org/) in R scripting. Any other use of scripts was only for preliminary work. All final data was both extracted and verified manually. We have therefore rewritten the relevant lines, since the wording was misleading. We didn’t make use of more scripts because everyone in the team did not have programming skills, and the data was amenable to manual handling.

**- - - - - - - - - - - - - - - - - - - - - - - - - - - - - - - - - - - -**

R4.21 pg 6 lines 148-149: What does 'pivotal' mean in this context?

**Response:** ‘Pivotal trials’ is a standard term. Based on query R1.5, we have described ‘pivotal’ trials in the Materials and Methods section.

**- - - - - - - - - - - - - - - - - - - - - - - - - - - - - - - - - - - -**

R4.22 pg 7 lines 159-169: This paragraph is really difficult to follow due to all the acronyms. Some of these may need spelling out.

**Response:** Mostly, we have used standard acronyms here. However we have generally reduced the number of acronyms in the manuscript, and that includes one of those (SI, for Study identifier) used in these lines. (Please also see our response to R4.3, above.)

**- - - - - - - - - - - - - - - - - - - - - - - - - - - - - - - - - - - -**

R4.23 pg 7 line 180: What did you do with the remaining 220 studies?

**Response:** Since no matches were found for the 220 studies, they could not be taken further for analysis. They were filtered out. A line to this effect has been added in the manuscript.

**- - - - - - - - - - - - - - - - - - - - - - - - - - - - - - - - - - - -**

R4.24 pg 13 lines 243-245: Why did you limit the orphan indications? It is maybe because of 'the challenge of examining the MedRs' but I'm not sure why this is relevant to the research question. I'm not sure too that I would start on limitations in the discussion section - you have a separately limitations section on pg 15, lines 309-313 so move most of this here.

**Response:** We have now discussed why we have studied RDs in the Introduction. We have also moved the discussion on limitations from Discussion to Limitations.

**- - - - - - - - - - - - - - - - - - - - - - - - - - - - - - - - - - - -**

R4.25 pg 14 lines 267-272: The data quality discussion needs to engage more with the literature on this. Why is it important that N1 and N2 match? Why should the reader care about these discrepancies? And why is 17% 'a large number'?

**Response:** This has been done.

**- - - - - - - - - - - - - - - - - - - - - - - - - - - - - - - - - - - -**
