# Supplementary material for: Discrepancies between FDA documents and ClinicalTrials.gov for Orphan Drug-related clinical trial data
Source: PLOS Glob Public Health. 2022 Apr 22;2(4):e0000261. doi: 10.1371/journal.pgph.0000261 (PMC10021800; doi:10.1371/journal.pgph.0000261)
Supplement: Attachment — Submitted filename: Response to reviewer 1s comments.doc [file pgph.0000261.s008.doc]

1. Line 35:

Reviewer Comment: This part is different to the Abstract in the first page of this document.

Our response: The Abstract had undergone some changes in the last round of revisions, but we had forgotten to enter the revised Abstract in the system where the Abstract is entered separately. We have now ensured that the two versions of the Abstract are identical.

- - - - - - - -- - - - - - - - - - - - - - - - - - - - - - - - - - - - - - - - - - - - - - - -

2. Table 1.

Reviewer Comment: It is sugested that the table title let to identify its content even outside of the main text, for example: "Sources of Orphan Drug-related clinical trial data".

This recommendation could be considered for other table titles.

Our response:

This has been done for all the Tables.

- - - - - - - -- - - - - - - - - - - - - - - - - - - - - - - - - - - - - - - - - - - - - - - -

3. Line 148

Reviewer Comment: It is suggested to describe in this section why the authors chose 2008

Our response: Based on a query in the first round of revisions, this point was addressed in the paragraph starting Line 290.

- - - - - - - -- - - - - - - - - - - - - - - - - - - - - - - - - - - - - - - - - - - - - - - -

4. Line 221

Reviewer Comment: This paragraph contents the complete information that is within the table 2, it is suggested to sintetizes this part, and describe only the relevant findings and refer the reader to the table to find the rest of the information.

Our response: This has been done.

- - - - - - - -- - - - - - - - - - - - - - - - - - - - - - - - - - - - - - - - - - - - - - - -

5. Table 4

Reviewer Comment: It seems that table 4 do not add anything to the description above, maybe it is not necessary

Our response: Table 4 has been removed.

- - - - - - - -- - - - - - - - - - - - - - - - - - - - - - - - - - - - - - - - - - - - - - - -

6. Line 260

Reviewer Comment: This paragraph contents the complete information that is within the table 5, it is suggested to sintetizes this part, and describe only the relevant findings and refer the reader to the table to find the rest of the information.

Our response: This has been done.

- - - - - - - -- - - - - - - - - - - - - - - - - - - - - - - - - - - - - - - - - - - - - - - -

7. Table 7

Reviewer Comment: It seems that table 7 do not add anything to the description above, maybe it is not necessary

Our response: Table 7 has been removed.

- - - - - - - -- - - - - - - - - - - - - - - - - - - - - - - - - - - - - - - - - - - - - - - -
